# Serum creatinine-to-cystatin C ratio and 1-year mortality risk in advanced breast cancer patients: a multicenter retrospective cohort study

**DOI:** 10.3389/fnut.2025.1688477

**Published:** 2025-11-26

**Authors:** Huijie Deng, Jiacheng Yang, Guanyu Zheng, Biyao Zhang, Yuzhou Wang, Yuye Wu, Shufen Mo, Shengchao Huang, Yuanqi Zhang, Lixia Li

**Affiliations:** 1 Cancer Hospital, Affiliated Hospital of Guangdong Medical University, Zhanjiang, China; 2 Medical Oncology Department II, Central Hospital of Guangdong Nongken, Zhanjiang, China; 3 Department of Breast Surgery, Affiliated Hospital of Guangdong Medical University, Zhanjiang, China

**Keywords:** breast cancer, creatinine, cystatin C, creatinine–cystatin C, muscle mass, sarcopenia, mortality

## Abstract

**Background:**

Muscle wasting and sarcopenia in advanced breast cancer correlates with poor outcomes. The serum creatinine-to-cystatin C ratio (CCR) is a potential muscle mass biomarker, but its prognostic value in advanced breast cancer is unclear.

**Methods:**

This multicenter retrospective cohort study included 465 patients with stage III-IV breast cancer (2018–2023) receiving standard treatment. The creatinine-cystatin C ratio (CCR) was calculated based on baseline serum markers. The primary endpoint was the 1-year all-cause mortality rate, as assessed through medical records and follow-up. A multivariate Cox regression model was used to analyze the relationship between CCR and mortality, along with restricted cubic spline, Kaplan–Meier survival analysis, ROC curve, and subgroup analysis.

**Results:**

This study enrolled a total of 465 patients with stage III-IV breast cancer, with a median age of 52.0 (interquartile range [IQR], 47.0–60.0)years and a median creatinine-cystatin C ratio (CCR) of 1.0 (IQR, 0.8–1.2). The 1-year mortality rate among all patients was 26.2% (122/465), with a mortality rate of 18.1% (52/288) for stage III patients and 39.5% (70/177) for stage IV patients. Multivariate Cox proportional hazards regression analysis showed a significant negative association between CCR and 1-year all-cause mortality in breast cancer patients (adjusted HR = 0.68, 95% CI: 0.63–0.74, *p* < 0.001). Compared with the lowest quartile group (Q1), the highest quartile group (Q4) had a mortality risk reduction of 94% (HR = 0.06, 95% CI: 0.03–0.14, *p* < 0.001). Restricted cubic spline analysis confirmed a linear negative association between the two (P for non-linear = 0.178). The Kaplan–Meier survival curves showed a significantly higher 1-year all-cause mortality in the Q1 group (*p* < 0.0001). The area under the curve (AUC) for predicting 1-year mortality was 0.802 (95% CI: 0.756–0.849). Subgroup analysis revealed a significant interaction between CCR and chemotherapy (*p* = 0.013) and clinical stage (*p* < 0.001), while the negative correlation persisted in other subgroups. Sensitivity analysis using unadjusted data yielded consistent results (Q4 HR = 0.06, 95% CI: 0.02–0.14), confirming the robustness of the study conclusions.

**Conclusion:**

The serum creatinine-cystatin C ratio is an independent predictor of 1-year mortality risk in advanced breast cancer, with higher levels associated with significantly reduced mortality.

## 1 Introduction

The 2022 edition of Global Cancer Statistics reports that breast cancer has emerged as a significant global health concern, ranking as the second most prevalent malignant tumor worldwide (1). Over recent decades, its incidence and mortality rates have risen sharply, imposing a considerable burden on public health systems. Breast cancer is a heterogeneous disease characterized by multiple subtypes, including hormone receptor-positive, HER2-positive, and triple-negative breast cancer, each exhibiting distinct clinical features, prognostic outcomes, and therapeutic options (2). Although advancements in early detection and systemic therapies have improved patient survival rates, the prognosis of advanced breast cancer (typically referring to stage III-IV disease) remains a major clinical challenge, with high mortality rates attributable to metastasis and systemic complications (3). Consequently, there is an urgent need to identify more accessible and precise prognostic biomarkers for stratifying advanced breast cancer patients at imminent risk of mortality, thereby enabling more aggressive interventions and personalized management approaches.

In recent years, various serology-based non-invasive indicators have been demonstrated to hold significant value in cancer prognosis assessment. For instance, inflammation-related markers such as the Systemic Immune-Inflammation Index (SII), Neutrophil-to-Lymphocyte Ratio (NLR), and Platelet-to-Lymphocyte Ratio (PLR) have been validated as significantly associated with patient survival outcomes across various malignancies, including gynecological cancers (4). These readily obtainable and cost-effective parameters provide convenient tools for clinical risk stratification. Furthermore, accumulating evidence indicates that systemic metabolic status profoundly influences cancer prognosis. For example, elevated fasting blood glucose and fatty liver disease have been identified as drivers of colorectal cancer (5), while increased non-invasive liver fibrosis scores are associated with malignant risk of thyroid nodules in populations with metabolic abnormalities (6). However, these markers primarily reflect systemic inflammatory status or metabolic dysregulation, rather than specifically indicating muscle mass. Given the significant implications of sarcopenia in advanced breast cancer and the fact that muscle tissue itself is a crucial metabolically active organ, developing serological markers capable of specifically assessing muscle reserves holds substantial clinical value.

One of the severe complications faced by patients with advanced breast cancer is cancer cachexia, a multifactorial metabolic syndrome characterized by involuntary weight loss, muscle wasting, and systemic inflammation (7). Sarcopenia, defined as the progressive and generalized loss of skeletal muscle mass and strength, represents a core manifestation of cancer cachexia. It has been identified as an independent adverse prognostic factor in various malignancies, including breast cancer. In breast cancer patients, sarcopenia is associated with treatment resistance, increased chemotherapy toxicity, and reduced progression-free survival (8–11). However, current clinical assessment tools for sarcopenia are limited by low sensitivity, operational complexity, high costs, and restricted applicability in specific populations. Consequently, sarcopenia is frequently underdiagnosed in routine clinical practice, highlighting an urgent need for simple and effective biomarkers to identify at-risk patients.

In recent years, the serum creatinine-to-cystatin C ratio (CCR) has gained attention as a potential surrogate marker for assessing muscle mass. Creatinine production primarily depends on skeletal muscle, whereas cystatin C mainly reflects glomerular filtration rate (GFR) and is less influenced by muscle mass. Thus, CCR theoretically offers a more specific reflection of muscle mass levels (9). Studies have demonstrated the clinical utility of CCR in high-risk populations for muscle wasting, including older adults (12), chronic kidney disease (CKD) (13), and cancer patients (14). As a low-cost, non-invasive indicator derived from routine blood tests, CCR provides a convenient approach for evaluating sarcopenia and predicting related adverse outcomes.

A large-scale retrospective clinical study (8) involving 3,060 patients with various malignancies (including breast cancer) demonstrated a significant association between serum creatinine to cystatin C ratio and survival outcomes in cancer patients. However, this study was exclusively conducted in a Korean population, with only 113 breast cancer cases (accounting for 3.7% of the cohort), and lacked breast cancer-specific stratified analyses. The generalizability of these findings to advanced breast cancer populations remains uncertain. To address this knowledge gap, we performed a retrospective analysis of electronic medical records from two tertiary medical centers in Zhanjiang, China, focusing specifically on stage III-IV breast cancer patients. This study was designed to explore the relationship between the creatinine-to-cystatin C ratio (CCR) and 1-year all-cause mortality in breast cancer patients, while evaluating the clinical validity of CCR as an independent serological prognostic marker for 1-year mortality risk.

## 2 Materials and methods

### 2.1 Study population

The primary focus of this study is to investigate the association between the creatinine-to-cystatin C ratio (a potential surrogate marker of sarcopenia) and all-cause mortality in breast cancer patients. In the initial planning stage, a sample size estimation was performed based on a binary exposure variable (sarcopenia vs. non-sarcopenia) for preliminary reference. According to the literature (9), the all-cause mortality rates during follow-up were 32% in the sarcopenia group and 17% in the non-sarcopenia group. In this study, with *α* = 0.05, power = 90%, a 1:1 case–control ratio, and two-sided testing, the sample size was calculated using the following formula:


$$n=\frac{{\left[z_{\alpha }\sqrt{2\mathrm{pq}}+z_{\beta }\sqrt{p_{1}(1-p_{1})+p_{2}(1-p_{2})}\right]}^{2}}{{\left(p_{1}-p_{2}\right)}^{2}},$$

yielding *n* = 277 cases. Considering the 1:1 grouping, 171 subjects were required for each of the sarcopenia and non-sarcopenia groups. Accounting for a 10% loss to follow-up and refusal rate, a minimum of 380 subjects were ultimately required for this study. It is important to note that the final statistical analysis employed a more robust and informative approach, treating CCR as both a continuous variable and in quartiles, rather than using a binary cutoff. Although the initial calculation was based on a different analytical premise, the final enrolled cohort of 465 patients exceeded the minimum sample size requirement. Furthermore, a post-hoc power analysis was conducted using PASS software (version 15) based on the observed effect size (adjusted HR = 0.68 per 10-unit CCR), total sample size (*N* = 465), and number of events (*n* = 122). This analysis confirmed that the study achieved a statistical power of >90% for detecting the observed association, thus validating the adequacy of the final sample size (15).

**Figure 1:**
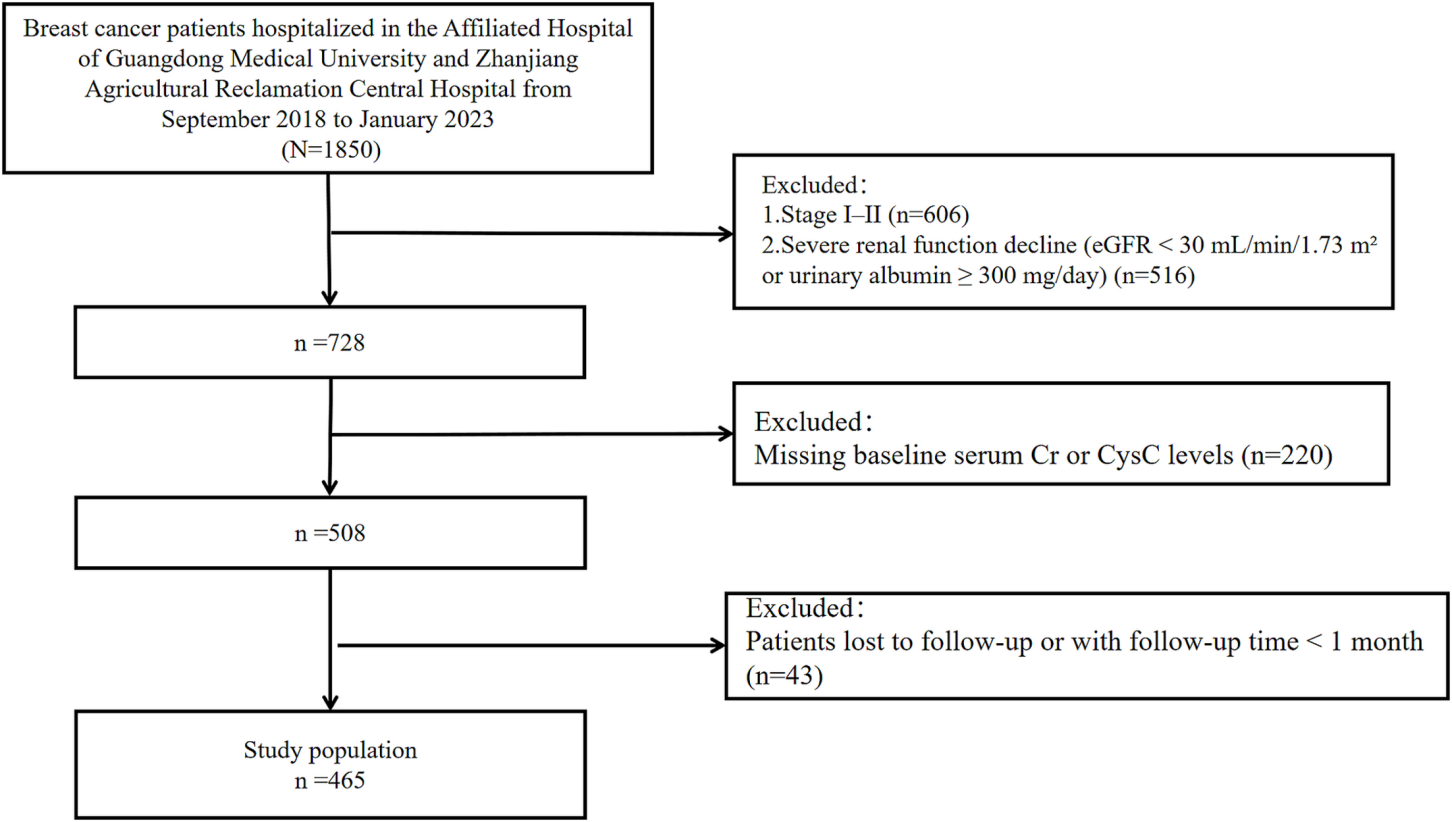
Flow diagram of the study.

This study retrospectively collected clinical data and follow-up outcomes from 465 breast cancer patients hospitalized at the Affiliated Hospital of Guangdong Medical University and Central Hospital of Guangdong Nongken between September 2018 and January 2023 (Figure 1). The patients of the Affiliated Hospital of Guangdong Medical University were identified from a breast cancer specific database from the well-established big-data intelligence platform at the Affiliated Hospital of Guangdong Medical University. It is a big-data intelligence platform at affiliated hospital of Guangdong medical university that enables real-time organizing, linking, and structuring data from a number of clinical business systems and thus allows health care providers to perform multidimensional big-data queries. This study followed the guidelines of the Declaration of Helsinki and obtained ethical approval from the Ethics Committee at the Affiliated Hospital of Guangdong Medical University (LY2024-09-012). Due to the retrospective study design, informed consent was waived for all participants.

### 2.2 Data collection

Demographic and laboratory data were obtained from electronic medical records. Baseline demographic and anthropometric data included age, sex, height, weight, estrogen receptor (ER) status, progesterone receptor (PR) status, human epidermal growth factor receptor 2 (HER2) status, breast cancer stage, treatment regimen, and tumor size. Body mass index (BMI) was determined by dividing an individual’s weight (in kilograms) by the square of their height (in meters), expressed as kg/m^2^. Laboratory parameters were collected from the first measurement after hospital admission (16), including serum creatinine (SCr), cystatin C (CysC), estimated glomerular filtration rate (eGFR), and prealbumin (PA). The Chronic Kidney Disease Epidemiology Collaboration (CKD-EPI) creatinine equation was used to calculate the eGFR (17). In this study, the creatinine-to-cystatin C ratio (CCR) was calculated using the following formula:


$$\mathrm{CCR}=\frac{\text{Serum creatinine}(\frac{\mu \mathrm{mol}}{L})/88.4}{\text{Serum cystatin}\;C\;(\mathrm{mg}/L)}.$$

The division by the factor of 88.4 is to convert the units of serum creatinine from μmol/L to mg/dL, as the conventional unit for serum creatinine in the CCR calculation is mg/dL (1 mg/dL = 88.4 μmol/L). This unit conversion ensures consistency with established methodologies and previous literature (18).

ER and PR status were determined according to the American Society of Clinical Oncology (ASCO)/College of American Pathologists (CAP) guidelines, where immunohistochemical (IHC) staining of ≥1% of nuclei was considered positive, while <1% was classified as negative (19–21). HER2 positivity was defined as IHC 3+ staining, whereas IHC 0 or 1 + was classified as negative. For equivocal cases (IHC 2+), HER2 gene amplification was further evaluated using *in situ* hybridization (ISH), with ISH positivity confirming HER2-positive status.

Breast cancer subtypes were classified based on hormone receptors (HRs) and HER2 expression as follows: HR+/HER2−, ER-positive or PR-positive, and HER2-negative; HR+/HER2+: ER-positive or PR-positive, and HER2-positive; HR−/HER2+: ER-negative, PR-negative, and HER2-positive; HR−/HER2−: ER-negative, PR-negative, HER2-negative, a subtype also known as triple-negative breast cancer (TNBC) (21). Tumor staging followed the 8th edition of the American Joint Committee on Cancer (AJCC) guidelines (22).

### 2.3 Clinical outcome

Patients were followed until loss to follow-up, death, or January 31, 2023. The primary outcome was 1-year all-cause mortality. Follow-up data were obtained through hospital records, outpatient visits, and telephone interviews. Survival time was calculated from the date of breast cancer diagnosis to the occurrence of death, loss to follow-up, or the study cutoff date, whichever came first.

### 2.4 Statistical analyses

All patients were categorized into four groups (Q1, Q2, Q3, Q4) based on quartiles of the creatinine-cystatin C ratio (CCR). Missing covariates were imputed using multiple imputation by chained equations (MICE). Continuous variables were expressed as mean ± standard deviation or median with interquartile range (IQR), and intergroup comparisons were performed using the Kruskal-Wallis test. Categorical variables were described as frequencies (percentages), with intergroup comparisons conducted using the chi-square test or Fisher’s exact test. The association between CCR and 1-year all-cause mortality in breast cancer patients was analyzed using Cox proportional hazards regression models. The CCR underwent standardized transformation before inclusion in the Cox proportional hazards regression model. To enhance numerical stability and clinical interpretability of the Cox regression model, we applied a linear scaling method by multiplying CCR values by 10 (i.e., CCR × 10). This ensures that unit changes in the analysis correspond to more reasonable hazard ratio (HR) values (i.e., a 10-unit change in the original CCR). Such scaling approaches are a standard practice in biomedical statistics for optimizing effect size presentation without altering the statistical essence or directionality of the results (23). Two adjusted models were employed: Model 1 adjusted for age and body mass index (BMI), while Model 2 further adjusted for tumor diameter, prealbumin (PA), targeted therapy, surgery, chemotherapy, radiotherapy, endocrine therapy, estrogen receptor (ER), progesterone receptor (PR), human epidermal growth factor receptor 2 (HER2), Ki67 index, and clinical stage. The covariates included in Model 2 were selected *a priori* based on their established prognostic value in breast cancer from the existing literature (8, 24), with the aim of evaluating the independent association of CCR with mortality after accounting for these key clinical, pathological, and therapeutic factors. Effect sizes were presented as hazard ratios (HR) with 95% confidence intervals (95% CI), and trend tests were performed using the Wald test for continuous variables. Receiver operating characteristic (ROC) curves were plotted to evaluate the predictive performance of the models for 1-year mortality in breast cancer patients. The area under the ROC curve (AUC) and its 95% CI were calculated, with AUC ≥ 0.7 indicating moderate or better predictive ability. Restricted cubic spline (RCS) plots were used to explore the relationship between the creatinine-to-cystatin C ratio and 1-year mortality. Survival data were described using the Kaplan–Meier method, and survival curves were compared using the log-rank test. Furthermore, to address the potential role of certain adjusted variables (e.g., clinical stage, surgery) as mediators on the causal pathway between CCR and mortality, we additionally conducted *post hoc* mediation analyses. A *p*-value <0.05 was considered statistically significant. All statistical analyses were performed using R software version 4.4.2. Additionally, a *post hoc* power analysis for the primary association (CCR as a continuous variable in the fully adjusted Model 2) was performed using PASS software (version 15).

## 3 Results

### 3.1 Baseline characteristics of patients

The study ultimately included 465 patients with advanced breast cancer (Table 1). The overall population was predominantly middle-aged and elderly, with a median age of 52.0 (IQR: 47.0–60.0) years. Compared with the lowest CCR group (Q1), patients in the highest CCR group (Q4) were younger (median age: 49.0 vs. 55.0 years), had a higher BMI (22.6 vs. 21.6 kg/m^2^), exhibited earlier clinical staging (proportion of stage III: 69.2% vs. 46.6%), and demonstrated higher HER2 positivity (56.4% vs. 40.5%), HR+/HER2 + subtype prevalence (49.6% vs. 27.6%), and surgical intervention rates (92.3% vs. 83.6%). Conversely, the CCR Q1 group showed a higher proportion of stage IV disease (53.4% vs. 30.8%), TNBC subtype (11.2% vs. 7.7%), and non-surgical management (16.4% vs. 7.7%). However, no significant differences were observed across CCR quartile groups regarding tumor diameter, serum creatinine levels, cystatin C levels, PA levels, ER status, PR status, HER2 status, Ki67 expression levels, or treatment modalities (surgery, targeted therapy, chemotherapy, radiotherapy, endocrine therapy). Notably, while serum creatinine (median: Q1 = 60.0, Q2 = 60.0, Q3 = 59.0, Q4 = 59.4 μmol/L; IQR: 54.0–65.0) and cystatin C (median: Q1 = 0.6, Q2 = 0.6, Q3 = 0.6, Q4 = 0.7 mg/L; IQR: 0.6–0.8) levels showed no statistically significant differences across quartiles (*p* = 0.833 and *p* = 0.999, respectively), systematic trends were observed: cystatin C increased slightly from Q1 to Q4 (median: 0.6 vs. 0.7 mg/L), while creatinine remained relatively stable. This pattern reflects the narrow interquartile ranges (IQRs) of both markers, which limited the sensitivity of Kruskal-Wallis tests to detect subtle differences. Crucially, the CCR ratio (median: Q1 = 0.8, Q2 = 1.0, Q3 = 1.1, Q4 = 1.2; *p* < 0.001) amplified these relative changes, enabling effective stratification of muscle mass status.

**Table 1:** Baseline characteristics according to creatinine–cystatin C ratio quartile.

Variables	Total	The quartiles of the creatinine–cystatin C ratio				*p*-value
		Q1	Q2	Q3	Q4	
	(*N* = 465)	(*N* = 116)	(*N* = 115)	(*N* = 117)	(*N* = 117)	
CCR	1.0 (0.8, 1.2)	0.7 (0.6, 0.8)	0.9 (0.9, 1.0)	1.1 (1.1, 1.2)	1.4 (1.3, 1.5)	<0.001
Age, years	52.0 (47.0, 60.0)	55.0 (49.0, 62.2)	54.0 (48.0, 61.0)	53.0 (48.0, 59.0)	49.0 (43.0, 52.0)	<0.001
BMI, kg/m^2^	22.2 (20.3, 24.6)	21.6 (19.6, 23.5)	22.5 (20.7, 25.7)	22.4 (20.7, 24.8)	22.6 (20.4, 24.8)	0.015
Tumor diameter, cm	3.0 (2.0, 4.4)	3.4 (2.0, 5.0)	3.0 (2.0, 4.0)	3.0 (2.1, 4.0)	3.0 (2.0, 4.0)	0.761
Clinical stage						0.001
III	288 (61.9)	54 (46.6)	75 (65.2)	78 (66.7)	81 (69.2)	
IV	177 (38.1)	62 (53.4)	40 (34.8)	39 (33.3)	36 (30.8)	
Cancer treatment						
Surgery						0.069
No	50 (10.8)	19 (16.4)	14 (12.2)	8 (6.8)	9 (7.7)	
Yes	415 (89.2)	97 (83.6)	101 (87.8)	109 (93.2)	108 (92.3)	
Chemotherapy						0.782
No	83 (17.8)	17 (14.7)	22 (19.1)	22 (18.8)	22 (18.8)	
Yes	382 (82.2)	99 (85.3)	93 (80.9)	95 (81.2)	95 (81.2)	
Target therapy						0.604
No	320 (68.8)	80 (69)	76 (66.1)	78 (66.7)	86 (73.5)	
Yes	145 (31.2)	36 (31)	39 (33.9)	39 (33.3)	31 (26.5)	
Endocrine therapy						0.862
No	330 (71.0)	84 (72.4)	78 (67.8)	84 (71.8)	84 (71.8)	
Yes	135 (29.0)	32 (27.6)	37 (32.2)	33 (28.2)	33 (28.2)	
Radiotherapy						0.2
No	308 (66.2)	84 (72.4)	71 (61.7)	72 (61.5)	81 (69.2)	
Yes	157 (33.8)	32 (27.6)	44 (38.3)	45 (38.5)	36 (30.8)	
Biomarker status						
ER						0.461
Negative	122 (26.2)	37 (31.9)	28 (24.3)	29 (24.8)	28 (23.9)	
Positive	343 (73.8)	79 (68.1)	87 (75.7)	88 (75.2)	89 (76.1)	
PR						0.229
Negative	204 (43.9)	53 (45.7)	41 (35.7)	56 (47.9)	54 (46.2)	
Positive	261 (56.1)	63 (54.3)	74 (64.3)	61 (52.1)	63 (53.8)	
HRE2						0.057
Negative	227 (48.8)	69 (59.5)	51 (44.3)	56 (47.9)	51 (43.6)	
Positive	238 (51.2)	47 (40.5)	64 (55.7)	61 (52.1)	66 (56.4)	
Ki67	40.0 (20.0, 60.0)	30.0 (13.8, 50.0)	40.0 (20.0, 70.0)	35.0 (20.0, 60.0)	40.0 (20.0, 50.0)	0.064
Molecular subtypes						0.007
HR+/HER2-	198 (42.6)	56 (48.3)	48 (41.7)	52 (44.4)	42 (35.9)	
HR+/HER2+	192 (41.3)	32 (27.6)	55 (47.8)	47 (40.2)	58 (49.6)	
HR-/HER2+	46 (9.9)	15 (12.9)	9 (7.8)	14 (12)	8 (6.8)	
TNBC	29 (6.2)	13 (11.2)	3 (2.6)	4 (3.4)	9 (7.7)	
Laboratory parameters						
Creatinine, umol/L	59.4 (52.0, 69.0)	60.0 (52.2, 70.0)	60.0 (50.0, 68.1)	59.0 (54.0, 69.0)	59.4 (51.0, 68.0)	0.833
Cystatin C, mg/L	0.6 (0.5, 0.8)	0.6 (0.5, 0.8)	0.6 (0.5, 0.8)	0.6 (0.5, 0.8)	0.7 (0.5, 0.8)	0.999
eGFR, ml/min	101.4 (90.2, 109.5)	103.4 (95.5, 112.5)	101.5 (88.7, 107.4)	98.4 (86.8, 108.8)	101.6 (90.2, 109.1)	0.02
PA, mg/L	262.5 (218.3, 301.7)	265.2 (228.0, 297.2)	252.2 (210.4, 299.1)	267.6 (223.5, 309.3)	262.7 (218.3, 298.6)	0.549

Data presented as median (interquartile range) for continuous variable and *n* (%) for categorical variables. The median values of serum creatinine and cystatin C were expressed by the interquartile intervals in parentheses (Q1, Q2, Q3, Q4). It shows the range of the SCr/CysC ratio for each quartile. CCR, creatinine−cystatin C ratio; BMI, Body Mass Index; ER, Estrogen Receptor; PR, Progesterone Receptor; HER2, Human Epidermal growth factor Receptor 2; eGFR, Glomerular filtration rate; PA, Prealbumin.

### 3.2 Multivariate analysis of CCR and 1-year all-cause mortality

To evaluate the association between the creatinine-cystatin C ratio and 1-year all-cause mortality, we constructed three Cox proportional hazards models (Table 2): Unadjusted model, No covariates were adjusted; Model 1, Adjusted for age and BMI based on the unadjusted model; Model 2, Further adjusted for tumor diameter, PA, targeted therapy, surgery, chemotherapy, radiotherapy, ER, PR, HER2, endocrine therapy, Ki67, and clinical stage in addition to Model 1. When analyzed as a continuous variable, each 10-unit increase in CCR was associated with a 33% reduction in mortality risk in the unadjusted model (HR = 0.67, 95% CI: 0.62–0.72, *p* < 0.001), a 32% reduction in Model 1 (HR = 0.68, 95% CI: 0.63–0.73, *p* < 0.001), and a 32% reduction in Model 2 (HR = 0.68, 95% CI: 0.63–0.74, *p* = 0.001). When analyzed by quartiles, with Q1 (lowest CCR group) as the reference, the HR values for Q2, Q3, and Q4 showed a progressively decreasing trend. In the fully adjusted Model 2, the Q4 group exhibited a significantly lower mortality risk compared to Q1 (HR = 0.06, 95% CI: 0.03–0.14, *p* < 0.001). The trend test yielded *p*-values < 0.001 in all models, confirming a significant linear correlation between CCR and 1-year all-cause mortality.

**Table 2:** Multivariable-adjusted HR and 95% CI of the CCR associated with 1-year all-cause mortality.

Variable	Unadjusted model		Model 1		Model 2	
	HR (95% CI)	*p* value	HR (95% CI)	*p* value	HR (95% CI)	*p* value
CCR (per 10 U)	0.67 (0.62 ~ 0.72)	<0.001	0.68 (0.63 ~ 0.73)	<0.001	0.68 (0.63 ~ 0.74)	0.001
Q1 (<8.192)	Referent		Referent		Referent	
Q2 (8.192–10.171)	0.19 (0.12 ~ 0.3)	<0.001	0.18 (0.11 ~ 0.29)	<0.001	0.19 (0.11 ~ 0.32)	<0.001
Q3(10.171–12.4)	0.15 (0.09 ~ 0.25)	<0.001	0.15 (0.09 ~ 0.25)	<0.001	0.16 (0.09 ~ 0.27)	<0.001
Q4(≥12.4)	0.05 (0.03 ~ 0.12)	<0.001	0.05 (0.02 ~ 0.11)	<0.001	0.06 (0.03 ~ 0.14)	<0.001
*p* for trend	-	<0.001	-	<0.001	-	<0.001

Model 1: Adjusted for age ang BMI; Model 2: Adjusted for Age, BMI, Tumor diameter, PA, Target therapy, Surgery, Chemotherapy, Radiotherapy, ER, PR, HRE2, Endocrine therapy, Ki67, Clinical stage; CCR, creatinine−cystatin C ratio; CI, confidence interval; HR, hazard ratio.

To further investigate whether the strong independent association between CCR and mortality could be mediated through changes in clinical stage or treatment allocation, we performed a *post hoc* mediation analysis. The results, detailed in Table 3, demonstrated that while clinical stage was a statistically significant mediator (*p* < 0.001), its proportion of mediation was minimal (3.41%). In contrast, none of the treatment modalities (surgery, chemotherapy, radiotherapy, targeted therapy, or endocrine therapy) showed a significant mediating effect (all *p* > 0.05). This indicates that the protective association of higher CCR with reduced mortality is largely (>96%) a direct effect, independent of its potential influence on disease stage or the receipt of specific anticancer therapies.

**Table 3:** Mediating effects of clinical stage and treatment-related variables on the association between CCR and 1-year mortality in breast cancer.

Mediator variable	Exposure → Mediator path (CCR→M)	Mediator → Outcome path (M → 1-year mortality)	Indirect effect	Direct effect	Total effect	Proportion mediated
	β (95% CI), *p*-value	β (95% CI), *p*-value	Estimate (95% CI), *p*-value	Estimate (95% CI), P-value	Estimate (95% CI), P-value	(IE/TE)%
Clinical stage	−1.32(−2.03, −0.61), <0.001*	0.92(0.40,1.44), 0.005*	−0.03(−0.04, -0.00), < 0.001*	−0.68(−0.74, −0.61), <0.001*	−0.70(−0.76, −0.64), < 0.001*	3.41
Surgery	0.41(−0.71,1.53), 0.471	−1.16(−1.91, −0.40), 0.003*	−0.01(−0.04, 0.00), 0.280	−0.69(−0.75, −0.62), < 0.001*	−0.70(−0.76, −0.63), <0.001*	0.84
Radiotherapy	−0.30(−0.97, 0.38), 0.391	0.59(0.06, 1.12), 0.029*	−0.00(−0.01, 0.01), 0.800	−0.71(−0.76, −0.65), < 0.001*	−0.71(−0.76, −0.65), <0.001*	0.11
Chemotherapy	−0.59(−1.43, 0.24), 0.162	1.11(0.33,1.88), 0.005*	−0.01(−0.02, 0.01), 0.320	−0.70(−0.76, −0.64), < 0.001*	−0.71(−0.76, −0.65), < 0.001*	0.67
Targeted therapy	−0.45(−1.14, 0.25), 0.210	0.47(−0.08, 1.02), 0.094	−0.00(−0.01, 0.00), 0.480	−0.70(−0.76, −0.64), < 0.001*	−0.71(−0.76, −0.64), < 0.001*	0.15
Endocrine Therapy	−0.32(−1.02,0.38), 0.372	0.29(−0.26, 0.83), 0.307	−0.00(−0.01, 0.00), 0.760	−0.71(−0.76, -0.65), < 0.001*	−0.71(−0.76, −0.65), < 0.001*	0.01

CCR, creatinine-cystatin C ratio; CI, confidence interval.

* Indicates statistical significance (*p* < 0.05).

**Figure 2:**
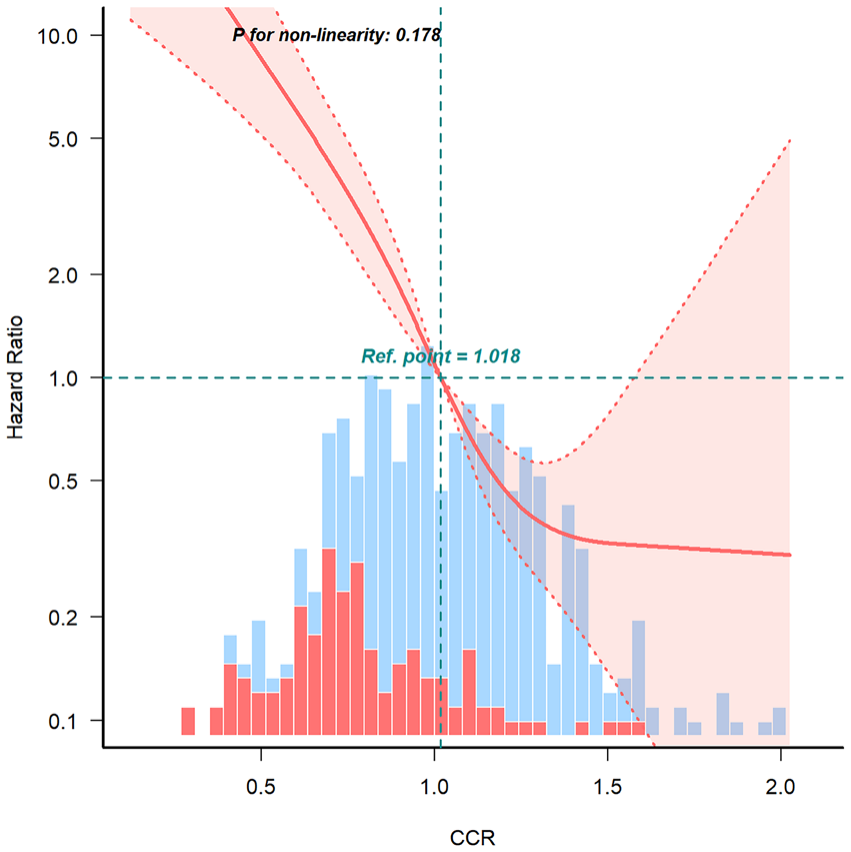
Restricted cubic spline curve for the association between creatinine–cystatin C ratio and 1-year all-cause mortality risk of breast cancer.

The results demonstrated a significant negative linear association between CCR and the risk of death in the fully adjusted RCS model (P for non-linearity = 0.178) (Figure 2). This finding suggests that as the creatinine-cystatin C ratio increases, the risk of 1-year all-cause mortality decreases.

**Figure 3:**
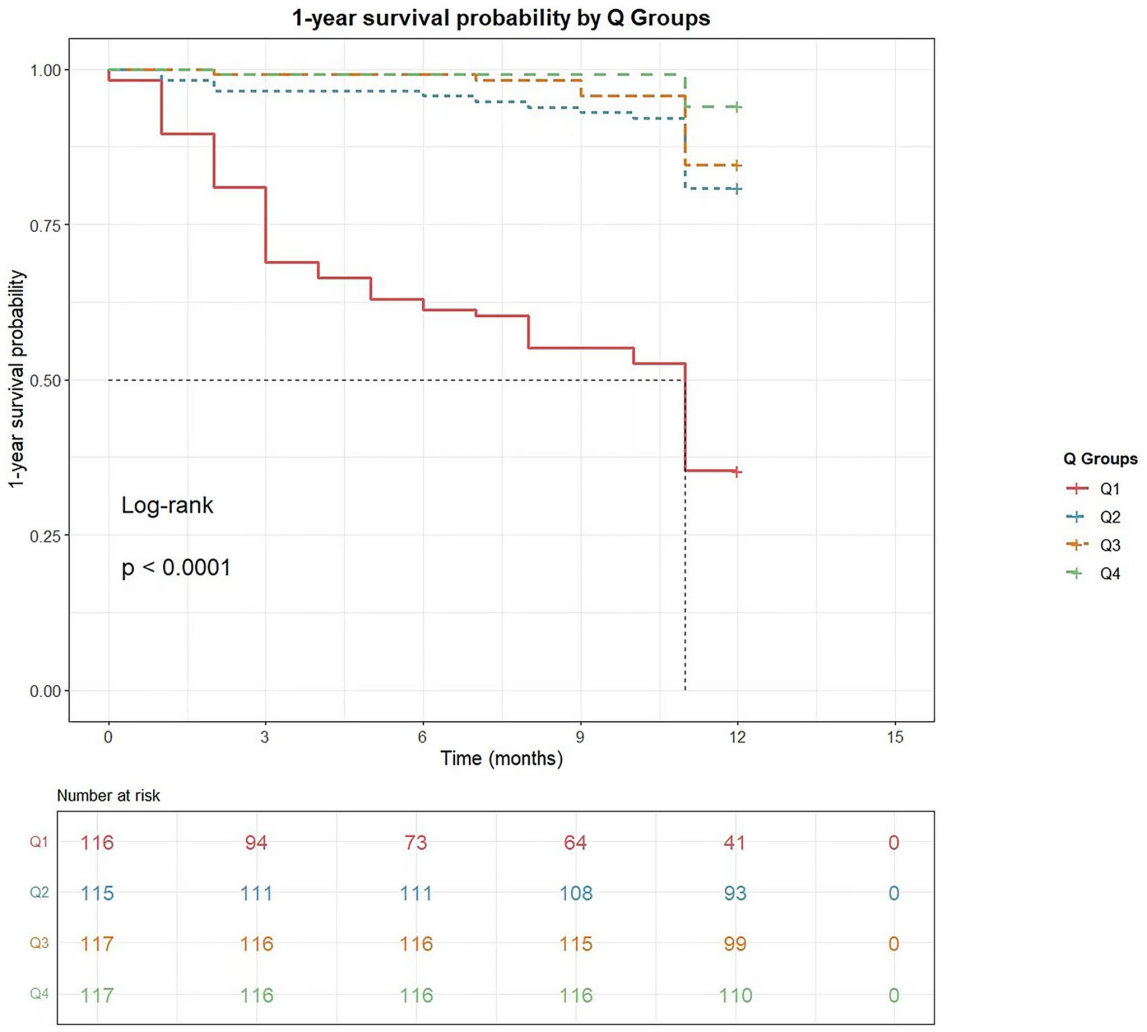
The cumulative survival probability within 1 year of cancer diagnosis based on creatinine-cystatin C ratio quartiles. Kaplan–Meier curves for 1-year survival stratified by creatinine-cystatin C ratio quartile.

Kaplan–Meier curves (Figure 3) demonstrated the 1-year overall survival probabilities of patients stratified by CCR quartiles. The log-rank test revealed significant differences in mortality risk among the four groups (*p* < 0.0001). The survival curves showed progressive separation with decreasing CCR levels, with the Q1 group (lowest CCR) exhibiting the highest 1-year all-cause mortality rate (lowest 1-year survival probability).

**Figure 4:**
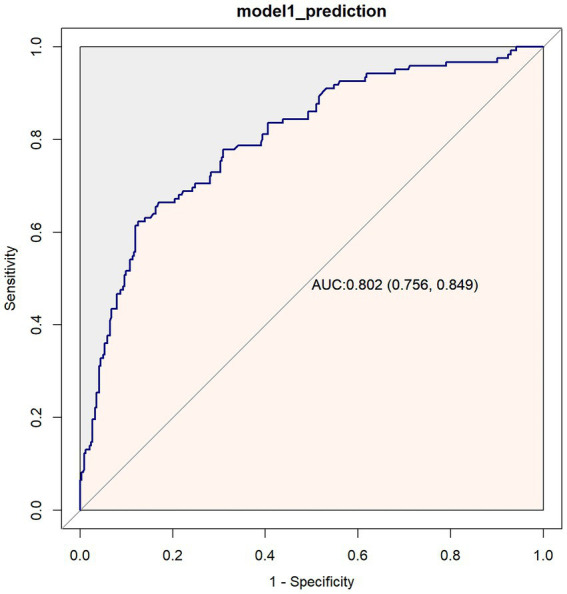
ROC curve for predicting 1-year mortality in breast cancer patients.

The predictive performance of the model for 1-year mortality in breast cancer patients was evaluated using receiver operating characteristic (ROC) curve analysis (Figure 4). The results demonstrated an area under the curve (AUC) of 0.802 (95%CI: 0.756–0.849), indicating good discriminative ability of the model.

### 3.3 Subgroup analyses

**Figure 5:**
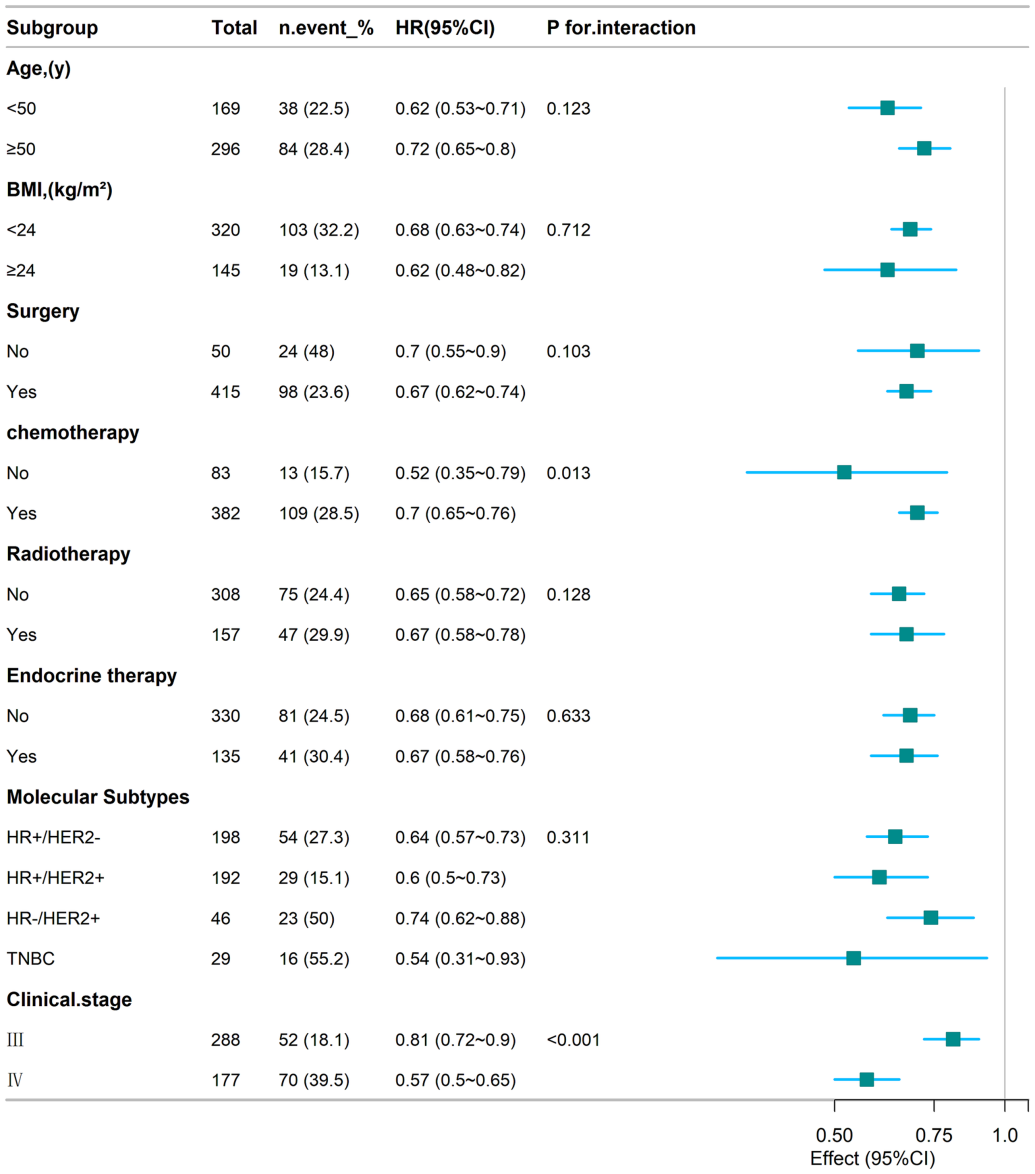
Subgroup analyses of the relationship between CCR and 1-year mortality in patients with breast cancer.

To explore whether there is heterogeneity in the correlation between the creatinine-cystatin C ratio (CCR) and 1-year mortality in breast cancer, this study stratified the groups according to age, BMI, surgery, chemotherapy, radiotherapy, endocrine therapy, molecular typing, and stage, and analyzed the interactions between them (Figure 5). The results showed that the interaction between CCR and mortality in breast cancer patients mainly differed between chemotherapy (P for interaction = 0.013) and clinical stage (P for interaction < 0.001). Additionally, in the subgroups stratified by age, BMI, surgery, radiotherapy, endocrine therapy, and molecular typing, the significant negative correlation between CCR and mortality in breast cancer patients persisted.

### 3.4 Sensitivity analyses

To assess the impact of missing value imputation on the results, we repeated all primary analyses using the original unimputed dataset (*n* = 465). Cox regression models for 1-year mortality were reconstructed with identical covariates. The unimputed analysis (Supplementary Table S2) revealed that in the fully adjusted model, each 10-unit increase in CCR was associated with a 31% reduction in 1-year all-cause mortality risk (HR = 0.69, 95% CI: 0.64–0.75) in advanced breast cancer patients. When analyzed by CCR quartiles, compared with the lowest quartile group (Q1), the highest quartile group (Q4) showed significantly lower 1-year mortality risk (HR = 0.06, 95% CI: 0.02–0.14). The RCS curve (Supplementary Figure 1) and subgroup analysis (Supplementary Figure 2) from unimputed data similarly replicated the negative linear association (P for non-linear = 0.195) and interaction patterns, with significant differences primarily observed for chemotherapy (P for interaction = 0.014) and clinical stage (P for interaction<0.001).

## 4 Discussion

This study represents the first investigation into the association between the creatinine/cystatin C ratio (CCR) and 1-year all-cause mortality among breast cancer patients in the Zhanjiang population of China. The results demonstrate that CCR serves as an independent predictor of 1-year mortality in advanced breast cancer patients, exhibiting a linear negative correlation. This association remained statistically significant even when CCR was analyzed by quartile stratification (Q1-Q4). Subsequent subgroup analyses confirmed the stability of this inverse correlation across diverse populations. Notably, this negative correlation was consistently observed across all molecular subtypes without significant interaction, suggesting that the prognostic value of CCR may be independent of breast cancer’s specific driver gene pathways (9, 24). This implies that CCR primarily reflects a “systemic state” of sarcopenia and inflammation driven by cancer cachexia (25), which uniformly impairs physiological reserve and survival outcomes across different pathological types. Receiver operating characteristic (ROC) curve analysis further indicated that CCR exhibits satisfactory predictive performance for 1-year mortality risk in breast cancer patients. These findings underscore the clinical value of CCR as an independent serological diagnostic marker for predicting 1-year mortality risk in breast cancer patients.

The apparent lack of significant differences in creatinine and cystatin C levels across CCR quartiles, despite the robust stratification of CCR itself, is biologically plausible and aligns with prior evidence. As a ratio, CCR integrates two complementary physiological parameters: creatinine (primarily derived from skeletal muscle) and cystatin C (reflecting glomerular filtration rate with minimal muscle dependence). Even minor shifts in their relative proportions—such as a slight rise in cystatin C (Q4 vs. Q1) coupled with stable creatinine—can produce meaningful CCR differences. This is consistent with studies in aging populations (12) and cancer patients (8), where CCR demonstrated superior predictive value for muscle mass and survival compared to either marker alone. Our findings further confirm that CCR’s strength lies not in absolute levels of its components, but in their relative ratio, which is more sensitive to subtle muscle mass variations.

The observed inverse correlation between CCR and breast cancer mortality can be explained by its biological characteristics as a surrogate marker of muscle mass. Creatinine (Cr) primarily originates from skeletal muscle metabolism, with its serum levels reflecting total muscle volume, whereas cystatin C (CysC) is secreted by all nucleated cells and remains relatively independent of muscle mass. Previous studies have consistently shown that sarcopenia is significantly associated with poorer clinical outcomes in breast cancer patients, including reduced treatment tolerance (24), increased complications (26), and shortened survival (27). A decreased Cr/CysC ratio directly reflects skeletal muscle depletion, which represents a core feature of cancer cachexia. Additionally, this negative correlation may also be linked to chronic inflammation-driven tumor progression. Studies (28, 29) have shown that pro-inflammatory cytokines (e.g., IL-6, CRP) stimulate nucleated cells to secrete cystatin C, leading to a decline in the Cr/CysC ratio. The inflammatory microenvironment further promotes angiogenesis, tumor migration, and immune evasion, directly contributing to increased mortality (30). In this study, patients in the low-CCR group (Q1) exhibited a higher proportion of stage IV disease (50%) and lower surgical intervention rates (85%). To quantitatively assess whether these observed associations with disease severity and treatment allocation underlie the link between CCR and mortality, we conducted a formal mediation analysis. The results (Table 3) revealed that clinical stage was a statistically significant but quantitatively minimal mediator, accounting for only 3.41% of the total effect. Crucially, none of the treatment modalities (surgery, chemotherapy, radiotherapy, targeted therapy, or endocrine therapy) demonstrated a significant mediating role. This pattern of findings suggests that while low CCR is associated with more advanced disease and fewer curative-intent treatments, the vast majority (>96%) of its strong association with mortality represents a direct effect. This implies that CCR primarily reflects a systemic, muscle-related physiological reserve whose depletion directly heightens mortality risk, largely independent of its correlation with tumor stage or specific treatment pathways. The linear relationship confirmed by restricted cubic spline analysis further supports that subtle variations in CCR can serve as a quantifiable indicator for dynamic monitoring of muscle status, providing a convenient clinical instrument for identifying high-risk patients early.

Notably, our findings extend prior work by Jung et al. (8), who reported CCR as a mortality predictor in cancer patients, to breast cancer specifically. While Jung et al. emphasized CCR’s prognostic value in the context of individual marker trends, our study demonstrates its utility even when creatinine and cystatin C levels show no statistical differences across quartiles—a critical nuance for biomarker validation in clinical practice. Previous studies have demonstrated that the creatinine/cystatin C ratio (CCR) effectively predicts mortality in patients with acute kidney injury (AKI) undergoing renal replacement therapy (31) and in cardiac surgery patients (32). Additionally, CCR serves as a significant predictor of hospitalization in chronic obstructive pulmonary disease (COPD) patients (33) and independently predicts functional outcomes in neurocritical care patients (34). Elevated cystatin C expression has been detected in colorectal cancer tissues compared to benign tissues (35), where increased cystatin C levels modulate cathepsin B—a lysosomal cysteine protease—promoting cancer invasion and basement membrane degradation. A similar phenomenon has been observed in breast cancer tissue samples (36). In patients with sarcopenia, low serum creatinine levels may lead to an overestimation of renal function, resulting in miscalculations of chemotherapy dosages. Consequently, these patients may receive higher-than-required drug doses, increasing the risk of chemotherapy-related toxicity. This elevated toxicity risk may partially explain the poorer survival observed in patients with lower CCR values (37). While cancer prognosis is primarily determined by disease-specific factors (e.g., tumor type, stage, grade, and genetic characteristics), general health indicators—such as clinical symptom presentation and nutritional status—also significantly influence outcomes (38). For instance, patients undergoing esophagectomy frequently experience weight loss and malnutrition due to altered dietary patterns. Prior research has shown that enteral nutritional support not only improves nutritional status and immune function but also preserves skeletal muscle mass (39). Home enteral nutrition has been reported to reduce malnutrition risk and postoperative complications following esophagectomy (40, 41), underscoring the importance of early sarcopenia screening. Building upon this evidence, a critical future goal is to determine if CCR’s utility can be translated from a prognostic indicator into a clinically actionable, modifiable treatment target. This would involve launching prospective, randomized controlled trials where patients with low CCR receive multimodal interventions aimed at reversing muscle loss, for example, through personalized nutritional support integrated with resistance exercise. The primary goal would be to determine if such targeted management can improve CCR values and, in turn, translate into tangible clinical benefits, including better treatment tolerance, improved quality of life, and prolonged overall survival. CCR is poised to become a key biomarker for both patient selection and efficacy evaluation in such studies.

The prognostic value of CCR identified in this study should be understood within the broader context of research on non-invasive serological biomarkers. In addition to CCR, various serological indicators have been demonstrated to hold value in prognostic assessment for breast cancer. For instance, inflammatory markers such as the Systemic Immune-Inflammation Index (SII) and Neutrophil-to-Lymphocyte Ratio (NLR) have been confirmed by multiple studies to be significantly associated with overall survival and progression-free survival in breast cancer patients (42). Unlike these inflammatory markers, the unique aspect of CCR lies in its direct reflection of muscle mass status rather than systemic inflammation. This characteristic enables CCR to provide prognostic information complementary to that from inflammatory markers, particularly in assessing cancer-related sarcopenia. In the field of gynecological cancers, similar studies have confirmed the prognostic value of non-invasive serological indicators. For example, in ovarian cancer patients, baseline NLR and PLR have been demonstrated to be independent prognostic factors (43). In endometrial cancer, serum albumin levels and inflammatory markers have also been confirmed to be associated with patient survival (44). Our research extends this field to the assessment of muscle mass and its metabolic associations in breast cancer, confirming the independent prognostic value of CCR as a surrogate marker of muscle mass in advanced breast cancer.

Previous studies by Jung et al. (8) indicated that a lower CCR was significantly associated with lower survival rates at 6 months and 1 year in cancer patients. Similarly, Sun et al. (45) demonstrated that gastric cancer patients with higher CCR exhibited prolonged survival compared to those with lower CCR. Ding et al. (46) further established an independent association between CCR and sarcopenia in gastrointestinal stromal tumor patients. Zheng et al. (47) also reported that CCR could effectively identify sarcopenia and serve as a prognostic marker for postoperative complications and long-term survival in esophageal cancer patients. In a study of 664 non-small cell lung cancer (NSCLC) patients, Chen et al. (48) found that CCR correlated with mortality in female patients. Gao et al. (10) observed that colorectal cancer (CRC) patients with higher CCR had significantly longer progression-free survival (PFS) and overall survival (OS) than those with lower CCR following surgical treatment. The present study further confirms a significant inverse correlation between CCR and 1-year all-cause mortality in stage III–IV breast cancer patients. After comprehensive adjustment for clinical and pathological confounders, every 10-unit increase in CCR was associated with a 32% reduction in mortality risk, while patients in the highest CCR quartile (Q4) exhibited a 94% lower mortality risk than those in the lowest quartile (Q1). Critically, our mediation analysis solidifies that this robust association is predominantly a direct effect, largely unmediated by disease stage or treatment decisions. These findings align with prior research across multiple malignancies and extend CCR’s prognostic utility to breast cancer, particularly offering a practical tool for resource-limited primary care settings lacking advanced imaging capabilities. Notably, the AUC of 0.802 underscores CCR’s clinical applicability as an easily accessible biomarker for risk stratification.

Despite the significant associations observed, our study has important limitations that should be noted. First, the analysis included only a limited set of confounders, while other potentially influential factors—such as patients’ underlying comorbidities and inflammatory markers—were not accounted for. Second, the creatinine/cystatin C ratio (CCR) in this study was based on a single measurement rather than dynamic longitudinal assessments, which may better reflect temporal changes in muscle mass and renal function. Third, given that cancer patients often experience malnutrition, serum creatinine levels may be influenced by nutritional status. Future research should examine how nutritional status and inflammatory processes—potentially measured through weight loss trajectories and CRP levels—mediate CCR’s association with mortality. Finally, as a retrospective study, our conclusions were derived from existing datasets with inherent constraints. Moreover, the study was not designed to explore the association between CCR and breast cancer-specific progression (e.g., progression-free survival), nor could it investigate whether improving CCR through interventions such as nutritional support would translate into better patient outcomes, which limits the insight into its potential as a modifiable risk factor. Future research should involve multicenter, multi-regional retrospective studies and prospective clinical trials to further elucidate the association between CCR and adverse outcomes in cancer patients. Such efforts would enhance the generalizability and robustness of our findings.

## Data Availability

The original contributions presented in the study are included in the article/Supplementary material, further inquiries can be directed to the corresponding authors.
