# Supplementary material for: Serum creatinine-to-cystatin C ratio and 1-year mortality risk in advanced breast cancer patients: a multicenter retrospective cohort study
Source: Front Nutr. 2025 Nov 26;12:1688477. doi: 10.3389/fnut.2025.1688477 (PMC12689403; doi:10.3389/fnut.2025.1688477)
Supplement: Supplementary file 1 [file Table_1.docx]

Table S1 Baseline characteristics according to creatinine–cystatin C ratio quartile

|  | | **The quartiles of the creatinine–cystatin C ratio** | | | |  |
| --- | --- | --- | --- | --- | --- | --- |
| **Variables** | **Total**  **(**N = 465) | **Q1**  **(**N = 116) | **Q2**  **(**N = 115) | **Q3**  **(**N = 117) | **Q4**  **(**N = 117) | **P-value** |
| **CCR** | 1.0 (0.8, 1.2) | 0.7 (0.6, 0.8) | 0.9 (0.9, 1.0) | 1.1 (1.1, 1.2) | 1.4 (1.3, 1.5) | <0.001 |
| **Age, years** | 52.0 (47.0, 60.0) | 55.0 (49.0, 62.2) | 54.0 (48.0, 61.0) | 53.0 (48.0, 59.0) | 49.0 (43.0, 52.0) | <0.001 |
| **BMI, kg/m^2^** | 22.2 (20.3, 24.6) | 21.6 (19.6, 23.5) | 22.5 (20.7, 25.7) | 22.4 (20.7, 24.8) | 22.6 (20.4, 24.8) | 0.015 |
| **Tumor diameter, cm** | 3.0 (2.0, 4.4) | 3.4 (2.0, 5.0) | 3.0 (2.0, 4.0) | 3.0 (2.1, 4.0) | 3.0 (2.0, 4.0) | 0.761 |
| **Clinical stage** |  |  |  |  |  | 0.001 |
| III | 288 (61.9) | 54 (46.6) | 75 (65.2) | 78 (66.7) | 81 (69.2) |  |
| IV | 177 (38.1) | 62 (53.4) | 40 (34.8) | 39 (33.3) | 36 (30.8) |  |
| **Cancer treatment** |  |  |  |  |  |  |
| **Surgery** |  |  |  |  |  | 0.069 |
| No | 50 (10.8) | 19 (16.4) | 14 (12.2) | 8 (6.8) | 9 (7.7) |  |
| Yes | 415 (89.2) | 97 (83.6) | 101 (87.8) | 109 (93.2) | 108 (92.3) |  |
| **Chemotherapy** |  |  |  |  |  | 0.782 |
| No | 83 (17.8) | 17 (14.7) | 22 (19.1) | 22 (18.8) | 22 (18.8) |  |
| Yes | 382 (82.2) | 99 (85.3) | 93 (80.9) | 95 (81.2) | 95 (81.2) |  |
| **Target therapy** |  |  |  |  |  | 0.604 |
| No | 320 (68.8) | 80 (69) | 76 (66.1) | 78 (66.7) | 86 (73.5) |  |
| Yes | 145 (31.2) | 36 (31) | 39 (33.9) | 39 (33.3) | 31 (26.5) |  |
| **Endocrine therapy** |  |  |  |  |  | 0.862 |
| No | 330 (71.0) | 84 (72.4) | 78 (67.8) | 84 (71.8) | 84 (71.8) |  |
| Yes | 135 (29.0) | 32 (27.6) | 37 (32.2) | 33 (28.2) | 33 (28.2) |  |
| **Radiotherapy** |  |  |  |  |  | 0.2 |
| No | 308 (66.2) | 84 (72.4) | 71 (61.7) | 72 (61.5) | 81 (69.2) |  |
| Yes | 157 (33.8) | 32 (27.6) | 44 (38.3) | 45 (38.5) | 36 (30.8) |  |
| **Biomarker Status** |  |  |  |  |  |  |
| **ER** |  |  |  |  |  | 0.393 |
| Negative | 122 (26.5) | 37 (32.7) | 28 (24.6) | 29 (24.8) | 28 (24.1) |  |
| Positive | 338 (73.5) | 76 (67.3) | 86 (75.4) | 88 (75.2) | 88 (75.9) |  |
| **PR** |  |  |  |  |  | 0.24 |
| Negative | 203 (44.1) | 53 (46.9) | 41 (36) | 56 (47.9) | 53 (45.7) |  |
| Positive | 257 (55.9) | 60 (53.1) | 73 (64) | 61 (52.1) | 63 (54.3) |  |
| **HRE2** |  |  |  |  |  | 0.029 |
| Negative | 217 (48.1) | 69 (60) | 48 (43.6) | 52 (46) | 48 (42.5) |  |
| Positive | 234 (51.9) | 46 (40) | 62 (56.4) | 61 (54) | 65 (57.5) |  |
| **Ki67** |  |  |  |  |  | 0.064 |
|  | 40.0 (20.0, 60.0) | 30.0 (13.8, 50.0) | 40.0 (20.0, 70.0) | 35.0 (20.0, 60.0) | 40.0 (20.0, 50.0) |  |
| **Molecular subtypes** |  |  |  |  |  | 0.003 |
| HR+/HER2- | 187 (41.6) | 54 (47.8) | 45 (40.9) | 49 (43.4) | 39 (34.5) |  |
| HR+/HER2+ | 189 (42.1) | 31 (27.4) | 54 (49.1) | 47 (41.6) | 57 (50.4) |  |
| HR-/HER2+ | 45 (10.0) | 15 (13.3) | 8 (7.3) | 14 (12.4) | 8 (7.1) |  |
| TNBC | 28 ( 6.2) | 13 (11.5) | 3 (2.7) | 3 (2.7) | 9 (8) |  |
| **Laboratory parameters** |  |  |  |  |  |  |
| **Creatinine, umol/l** | 59.4 (52.0, 69.0) | 60.0 (52.2, 70.0) | 60.0 (50.0, 68.1) | 59.0 (54.0, 69.0) | 59.4 (51.0, 68.0) | 0.833 |
| **Cystatin C, mg/l** | 0.6 (0.5, 0.8) | 0.6 (0.5, 0.8) | 0.6 (0.5, 0.8) | 0.6 (0.5, 0.8) | 0.7 (0.5, 0.8) | 0.999 |
| **eGFR, ml/min** | 101.4 (90.2, 109.5) | 103.4 (95.5, 112.5) | 101.5 (88.7, 107.4) | 98.4 (86.8, 108.8) | 101.6 (90.2, 109.1) | 0.02 |
| **PA, mg/l** | 262.6 (218.1, 301.9) | 264.7 (223.2, 298.2) | 255.2 (209.9, 299.3) | 267.6 (223.5, 309.3) | 263.3 (218.3, 300.0) | 0.583 |
| Data presented as median (interquartile range) for continuous variable and n (%) for categorical variables  Note: The median values of serum creatinine and cystatin C were expressed by the interquartile intervals in parentheses (Q1, Q2, Q3, Q4). It shows the range of the SCr/CysC ratio for each quartile.  Abbreviations: CCR: creatinine–cystatin C ratio; BMI: Body Mass Index; ER：Estrogen Receptor; PR：Progesterone Receptor; HER2：Human Epidermal growth factor Receptor 2; eGFR: Glomerular filtration rate; PA: Prealbumin | | | | | | |
