# Supplementary material for: Serum creatinine-to-cystatin C ratio and 1-year mortality risk in advanced breast cancer patients: a multicenter retrospective cohort study
Source: Front Nutr. 2025 Nov 26;12:1688477. doi: 10.3389/fnut.2025.1688477 (PMC12689403; doi:10.3389/fnut.2025.1688477)
Supplement: Supplementary file 2 [file Table_2.docx]

**Table S2** Multivariable-adjusted HR and 95% CI of the CCR（per 10 U） quartiles associated with 1-year mortality

| **Variable** | **Unadjusted** | | **Model 1** | | **Model 2** | |
| --- | --- | --- | --- | --- | --- | --- |
|  | **HR (95% CI)** | **p value** | **HR (95% CI)** | **p value** | **HR (95% CI)** | **p value** |
| CCR  （per 10 U） | 0.67 (0.62~0.72) | <0.001 | 0.68 (0.63~0.73) | <0.001 | 0.69 (0.64~0.75) | 0.001 |
| Q1(<8.192) | Referent |  | Referent |  | Referent |  |
| Q2 (8.192–10.171) | 0.19 (0.12~0.3) | <0.001 | 0.18 (0.11~0.29) | <0.001 | 0.19 (0.11~0.34) | <0.001 |
| Q3(10.171–12.4) | 0.15 (0.09~0.25) | <0.001 | 0.15 (0.09~0.25) | <0.001 | 0.16 (0.09~0.28) | <0.001 |
| Q4(≥12.4) | 0.05 (0.03~0.12) | <0.001 | 0.05 (0.02~0.11) | <0.001 | 0.06 (0.02~0.14) | <0.001 |
| p for trend | 0.35 (0.29~0.44) | <0.001 | 0.35 (0.28~0.44) | <0.001 | 0.38 (0.3~0.47) | <0.001 |

Model 1: Adjusted for age ang BMI;

Model 2: Adjusted for Age, BMI, Tumor diameter, PA, Target therapy, Surgery, Chemotherapy, Radiotherapy, Endocrine therapy, ER, PR, HRE2, Ki67, Clinical stage;
