# Supplementary figures and images for: Serum creatinine-to-cystatin C ratio and 1-year mortality risk in advanced breast cancer patients: a multicenter retrospective cohort study
Source: Front Nutr. 2025 Nov 26;12:1688477. doi: 10.3389/fnut.2025.1688477 (PMC12689403; doi:10.3389/fnut.2025.1688477)

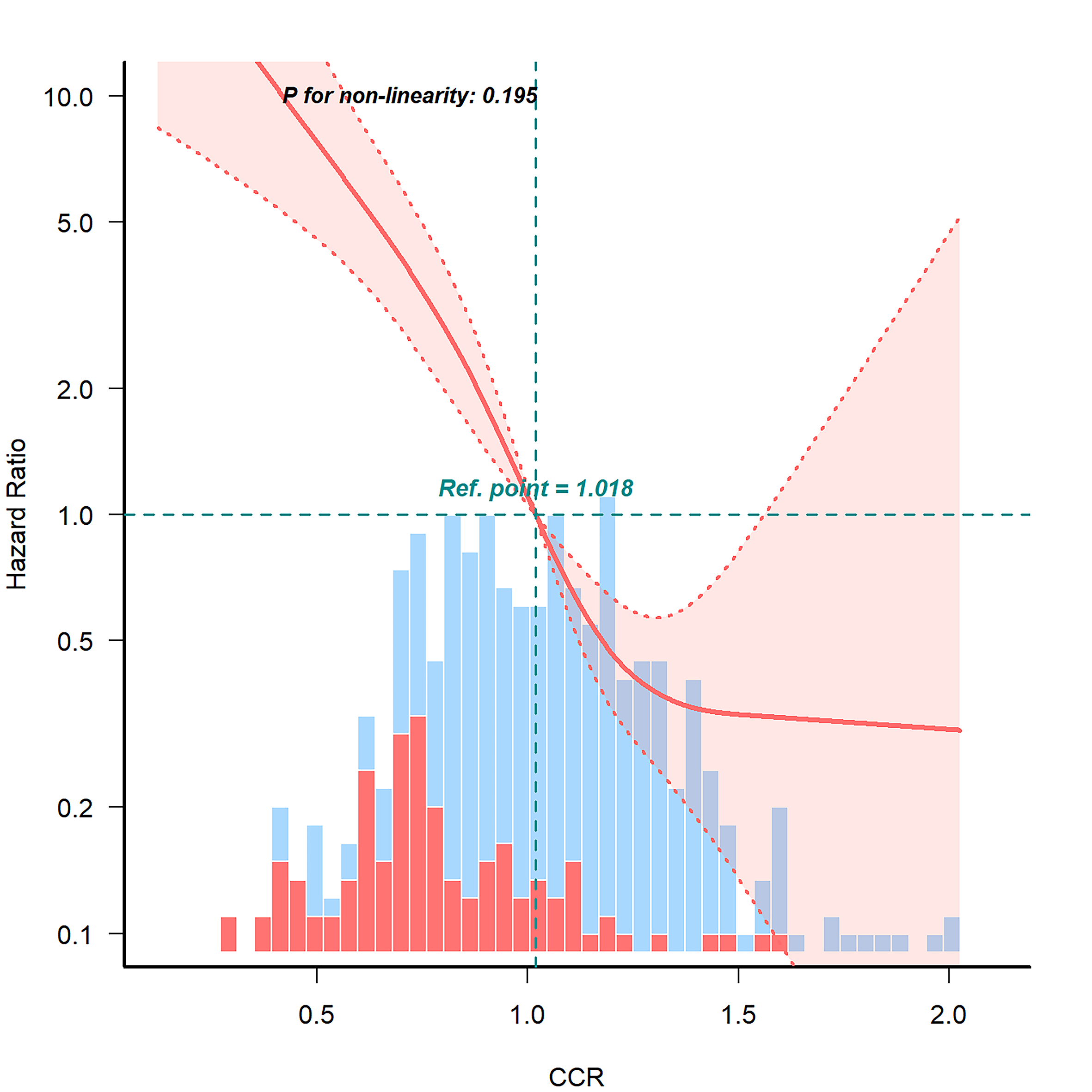

Supplement: Supplementary file 4 [file Image_1.tif]

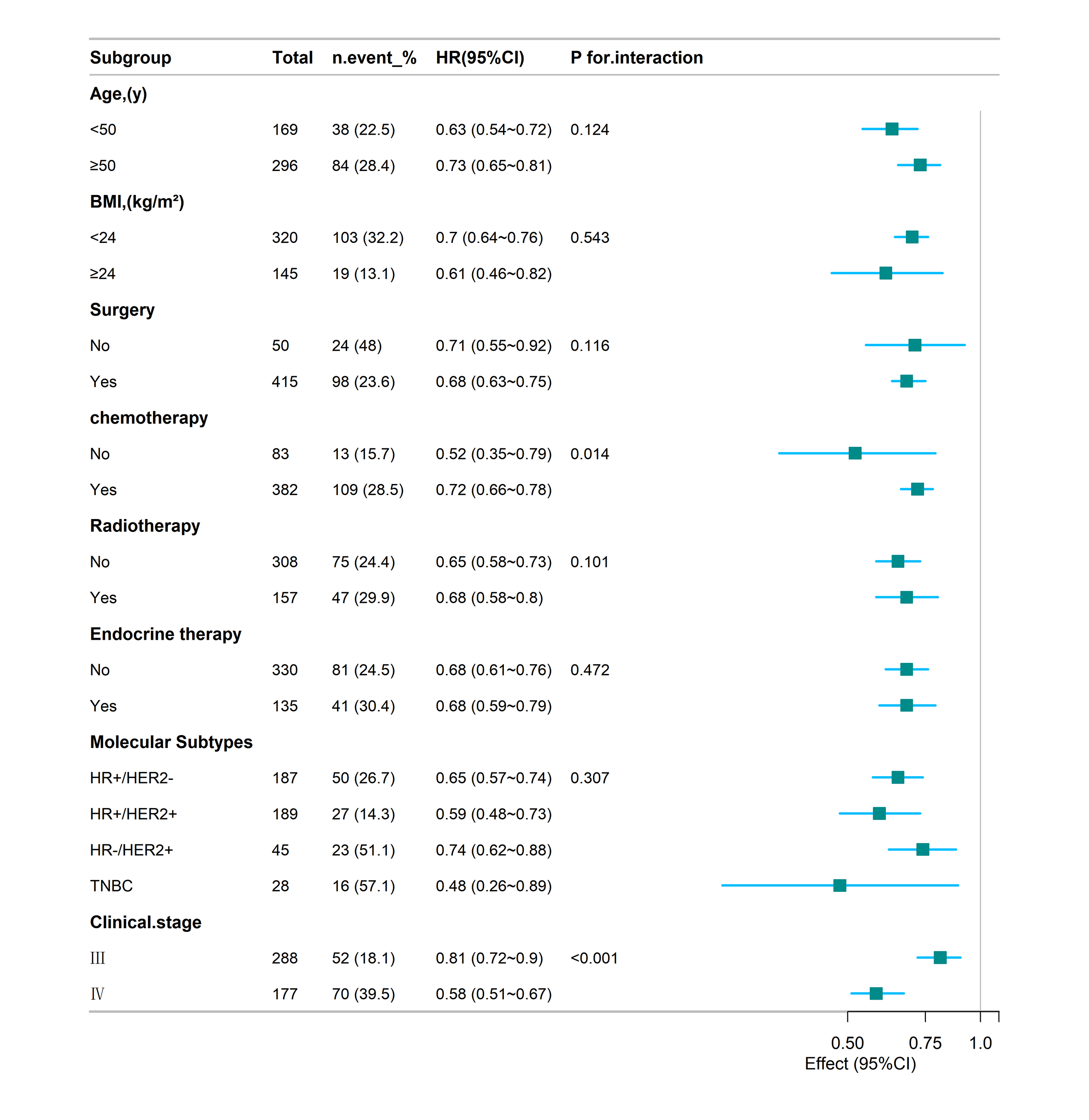

Supplement: Supplementary file 5 [file Image_2.tif]
